# Atypical atrial resetting with ventricular extrastimulus during tachycardia: What is the mechanism?

**DOI:** 10.1002/joa3.13126

**Published:** 2024-08-07

**Authors:** Takashi Kobari, Yoshiaki Kaneko, Shuntaro Tamura, Hiroshi Hasegawa, Yosuke Nakatani

**Affiliations:** ^1^ Department of Cardiovascular Medicine Gunma University Graduate School of Medicine Maebashi Japan

## Abstract

This atypical atrial resetting with ventricular extrastimulus delivered during supraventricular tachycardia is characterized by no capture of local ventricular deflection contralateral to the earliest atrial site and is a finding unmasking the presence of a nodoventricular pathway, the ventricular insertion of which is located apically, away from the mitral annulus.

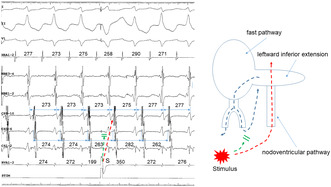

A 49‐year‐old man was admitted to our hospital for catheter ablation of narrow QRS supraventricular tachycardia. During an isoproterenol infusion, the tachycardia clinically documented was reproducibly induced by premature atrial stimulation (Figure 1A). The site of earliest atrial activation during tachycardia was found in the left posteroseptum (CS9‐10). A septal AH/ HA ratio during tachycardia was less than 1. The interval between the QRS onset and the earliest atrial activation was 91 ms. Overdrive stimulation from the right ventricular apex entrained the tachycardia, confirmed by (a) 1:1 capture of the atria, (b) an atrial activation sequence identical to that observed during tachycardia, and (c) continuation of the tachycardia upon cessation of entrainment (Figure 1B). The initial atrial (A) and ventricular (V) activation upon cessation of ventricular stimulation was a V‐A‐V sequence, excluding the diagnosis of atrial tachycardia (Figure 1B). The postpacing interval minus the tachycardia cycle (CL) at the site of stimulation, equals 67 ms (Figure 1B). Entrainment from the ventricle results in orthodromic capture of the His bundle (HB) with QRS fusion (Figure 1B). Moreover, a single premature ventricular stimulus (S) delivered during ongoing tachycardia, at the time of HB refractoriness, reproducibly reset the next atrial CL (Figure 2), excluding a diagnosis of AV nodal reentrant tachycardia.

**FIGURE 1:**
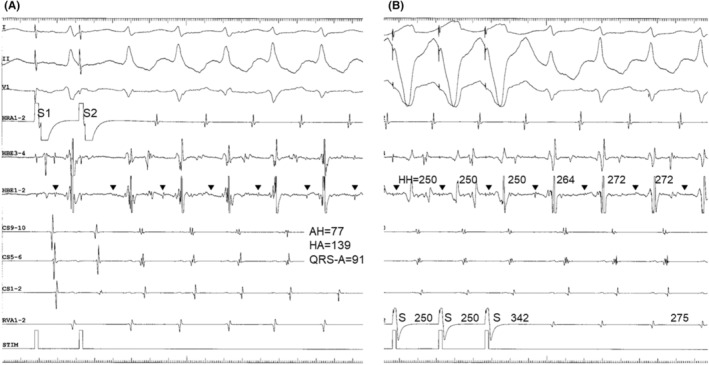
Induction of tachycardia by premature atrial stimulation (A) and return of tachycardia upon cessation of ventricular entrainment (B). (A) An S1‐S1 cycle length = 500 ms; an S1–S2 coupling interval = 240 ms. (B) An S‐S cycle length = 250 ms. Arrowheads indicate His bundle electrograms.

**FIGURE 2:**
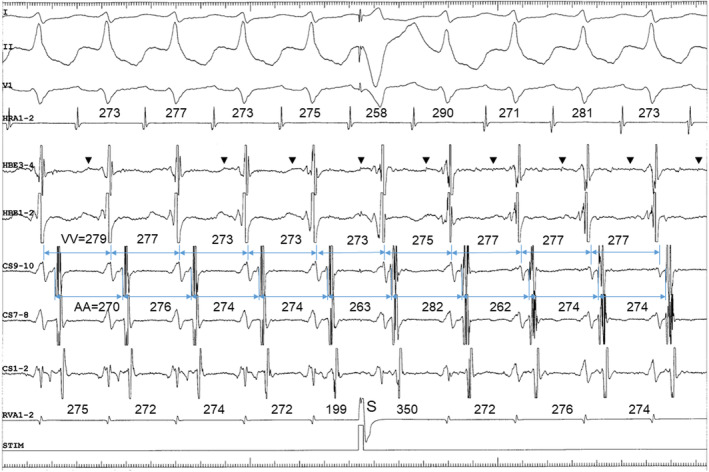
Atypical atrial preexcitation phenomenon. Numbers above lead HRA1‐2 indicate interatrial cycles. Bidirectional arrows and numbers above lead CS9‐10, magnified to closely inspect the far‐field ventricular electrograms, indicate interventricular cycles was constant.

All electrophysiological findings described earlier are consistent with orthodromic reentrant tachycardia using any type of accessory pathway as a regrograde limb. AV nodal pattern observed during parahisian pacing (Figure S1) does not completely exclude a possibility of the presence of posteroseptal AV accessory pathway. However, ventricular extrastimulation exhibited the absence of fusion of retrograde atrial activation over the fast and accessory pathways with a similar conduction time (Figure 3), inconsistent with the presence of AV accessory pathway. Also during ventricular overdrive stimulation, a retrograde conduction over the accessory pathway was manifested in atypical manner (Figure S2). Moreover, when comparing the AH interval measured during tachycardia with that during atrial stimulation at a similar CL within a few minutes, the former was 69 ms shorter than the latter (Figure 4), incompatible with the effects of the autonomic tone.^1^ Sustained antegrade conduction over the slow pathway is also unlikely because of no demonstration of dual AV nodal physiology. Accordingly, this observation suggests no participation of atria in the critical reentrant circuit that supports a diagnosis of orthodromic reentrant tachycardia using a concealed nodoventricular pathway (NVP) as the retrograde limb of the reentrant circuit. ^2^ Moreover, successful elimination of a retrograde conduction over the NVP was obtained by ablating at the earliest site of retrograde atrial activation in the left posteroseptal region, along the mitral annulus, suggesting a rare NVP connected with a left inferior AV nodal extension^3^ and with an atrial breakthrough in left posteroseptum (Figure S3). Retrograde manifestation via the NVP during ventricular stimulation may be probably because of a linking mechanism (Figure S4).

**FIGURE 3:**
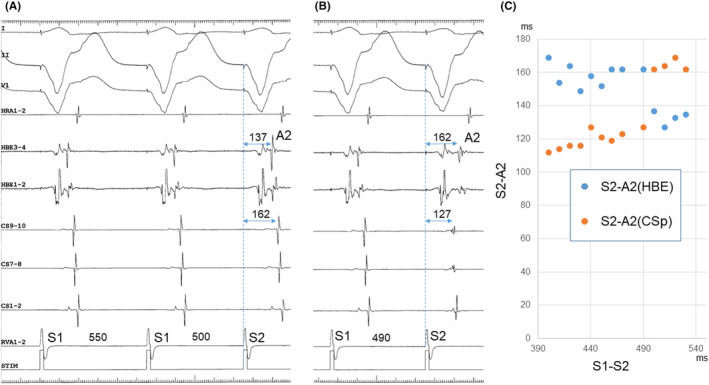
Atypical manifestation of retrograde conduction of premature ventricular stimuli over an accessory pathway. (A) Single premature ventricular stimuli delivered at ≥500 ms coupling intervals are conducted retrogradely over a fast pathway. (B) At S1‐S2 coupling intervals ≤490 ms, retrograde conduction switched to an accessory pathway, and the S‐atrial intervals measured after the premature stimuli at the proximal coronary sinus (S2‐A2(CSp)) became shorter in (B) than in (A), while the S‐atrial interval in the His bundle region (S2‐A2(HBE)) lengthened discontinuously. (C) Corresponding ventriculoatrial conduction curve. S2‐A2(HBE) and S2‐A2(CS) are shown by blue and brown dots, respectively.

**FIGURE 4:**
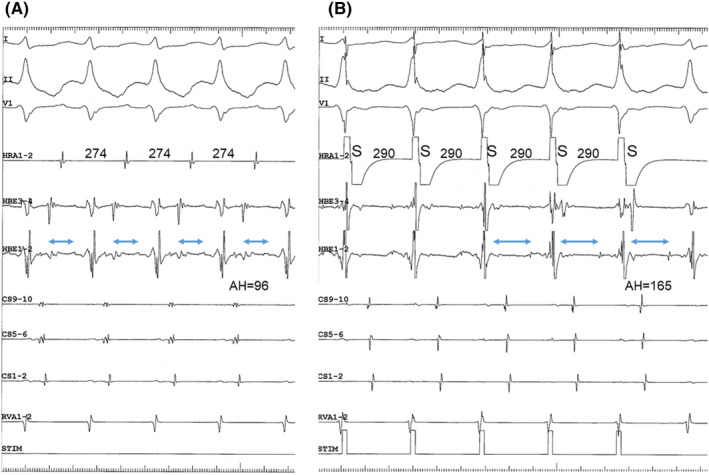
Atrio‐His interval (indicated by horizontal bidirectional arrows labeled above lead HBE1‐2) during tachycardia (A) versus during atrial overdrive stimulation (B). (A) The atrio‐His interval during tachycardia at a CL of 274 ms was 96 ms. (B) The atrio‐His interval during atrial overdrive stimulation at a slightly longer CL of 290 ms was 165 ms.

Interestingly, a meticulous inspection of the coronary sinus recordings during atrial preexcitation phenomenon revealed that the stimulus captured atria without capturing local ventricular deflection contralateral to the site of earliest atrial activation, inconsistent with the AV accessory pathway (Figure 2). This atypical atrial preexcitation is a novel finding unmasking the presence of a NVP, the ventricular insertion of which was located apically, away from the mitral annulus.

## FUNDING INFORMATION

None.

## CONFLICT OF INTEREST STATEMENT

None.

## ETHICS STATEMENT

Ethics approval to report this case was obtained from the institutional review board of Gunma University Hospital.

## PATIENT CONSENT STATEMENT

Informed consent was obtained from the patient for the purpose of this report.

## Permission to reproduce material from other sources

None.

## Supporting information


Data S1.

## Data Availability

The data that support the findings of this study are available on request from the corresponding author, YK.
