# Supplementary material for: Atypical atrial resetting with ventricular extrastimulus during tachycardia: What is the mechanism?
Source: J Arrhythm. 2024 Aug 7;40(5):1192–5. doi: 10.1002/joa3.13126 (PMC11474880; doi:10.1002/joa3.13126)
Supplement: Supplementary file 1 — Data S1. [file JOA3-40-1192-s001.docx]

**Supplemental figure 1**

**Absence of retrograde conduction over a nodoventricular pathway during parahisian pacing at an S-S cycle length of 500 ms**


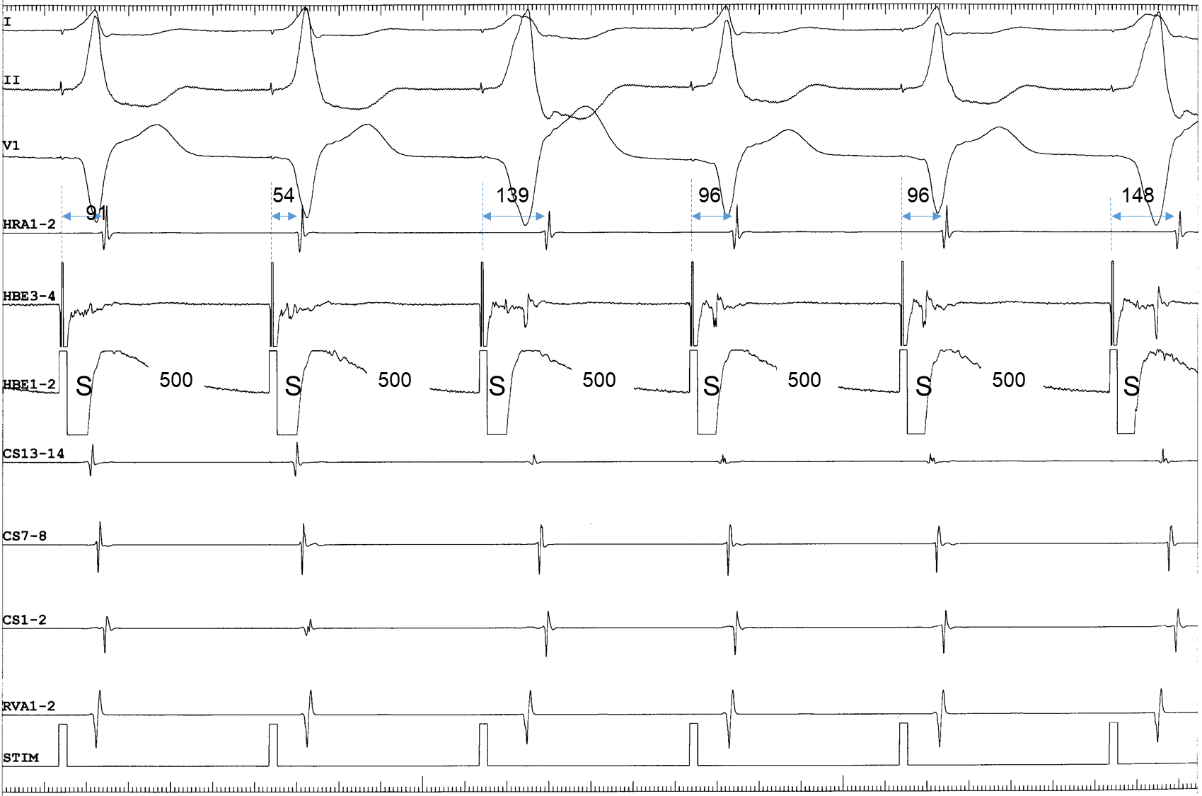


The 1st, 2nd, 4th and 5th QRS complexes are narrow, indicative of direct His bundle capture, while the 3rd and 6th QRS complexes are wide, consistent with ventricular myocardial capture. The 2nd cycle was excluded from this analysis because of direct atrial capture. The S-atrial intervals (bidirectional arrows above lead HRA1-2) associated with narrow QRS are shorter than when associated with a wide QRS, while the sequence of atrial activation, with the earliest site in the His bundle region (HBE1-2), is unchanged. This indicates that retrograde conduction occurred strictly over a fast AV nodal pathway. During ventricular stimulation at this CL, no retrograde conduction over a nodoventricular pathway was observed. I, II and V_1_ = leads of surface electrocardiogram; HBE1-2 and 3-4 = distal and proximal His bundle electrograms; CS1-2 to 9-10 = distal to proximal coronary sinus; RVA1-2 = right ventricular apex.

**Supplemental figure 2**

**Atypical retrograde conduction over a nodoventricular pathway during ventricular overdrive stimulation at an A) 400 and B) 350 ms S-S cycle length.**

**
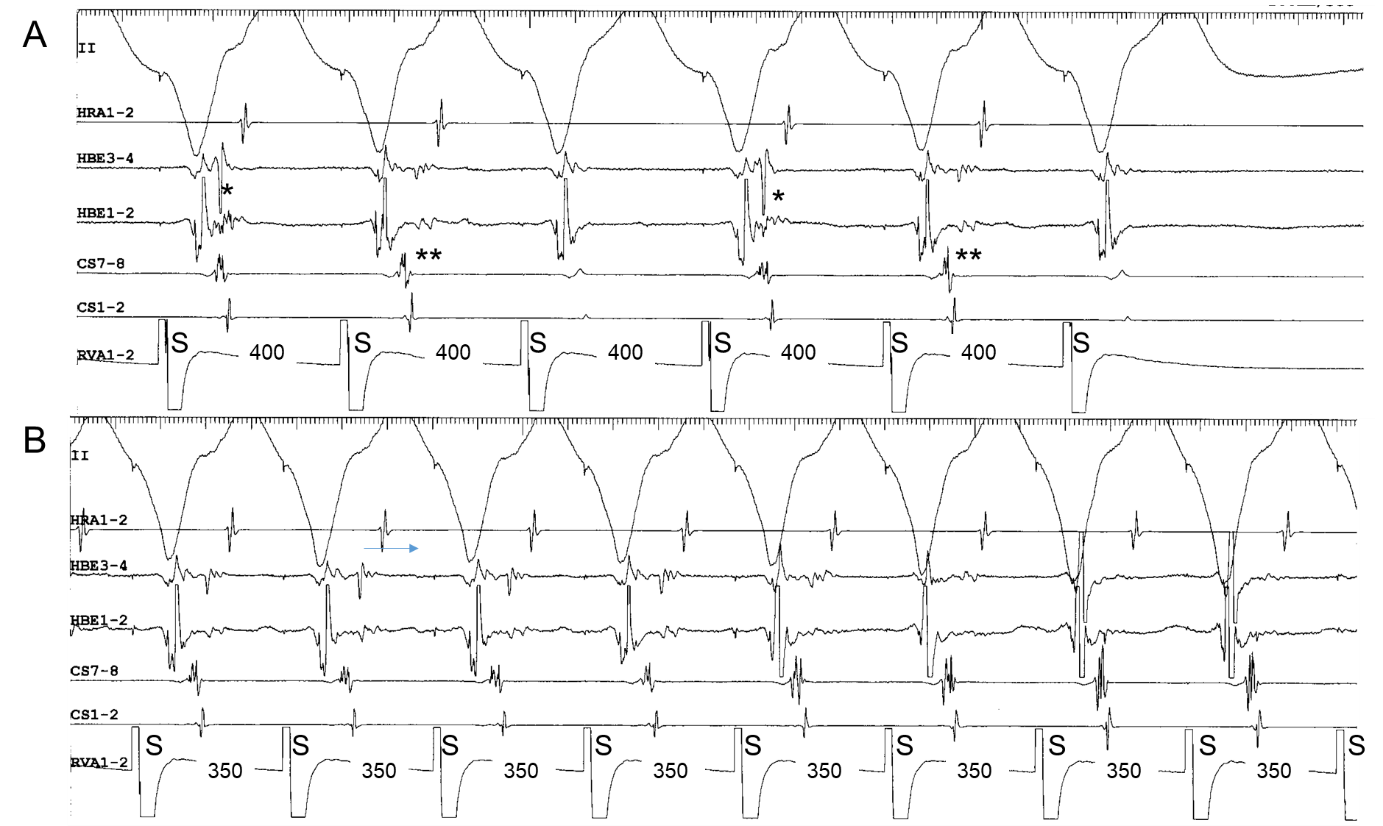
**

**A. SS CL = 400 ms:** Atypical 3:2 ventriculoatrial block. In the 1^st^ cycle, preceded by ventriculoatrial block, the earliest atrial activation was recorded in the His bundle region (HBE1-2;*), consistent with retrograde conduction over a fast pathway. In the 2^nd^ cycle, the earliest atrial activation was recorded at the proximal coronary sinus (CS7-8;**), indicative of retrograde conduction over a nodoventricular pathway.

**B. SS CL = 350 ms:** 3:2 retrograde conduction alternating over the fast and the nodoventricular pathways was replaced by retrograde conduction exclusively over the nodoventricular pathway, suggesting that retrograde conduction over that pathway did not merely depend on delayed conduction in the fast pathway. See supplemental figure 1 for other abbreviations.

**Supplemental figure 3**

**Successful ablation in the left posterior septal region (A and B) and the illustration of the reentrant conductive pathways during tachycardia (C)**

**
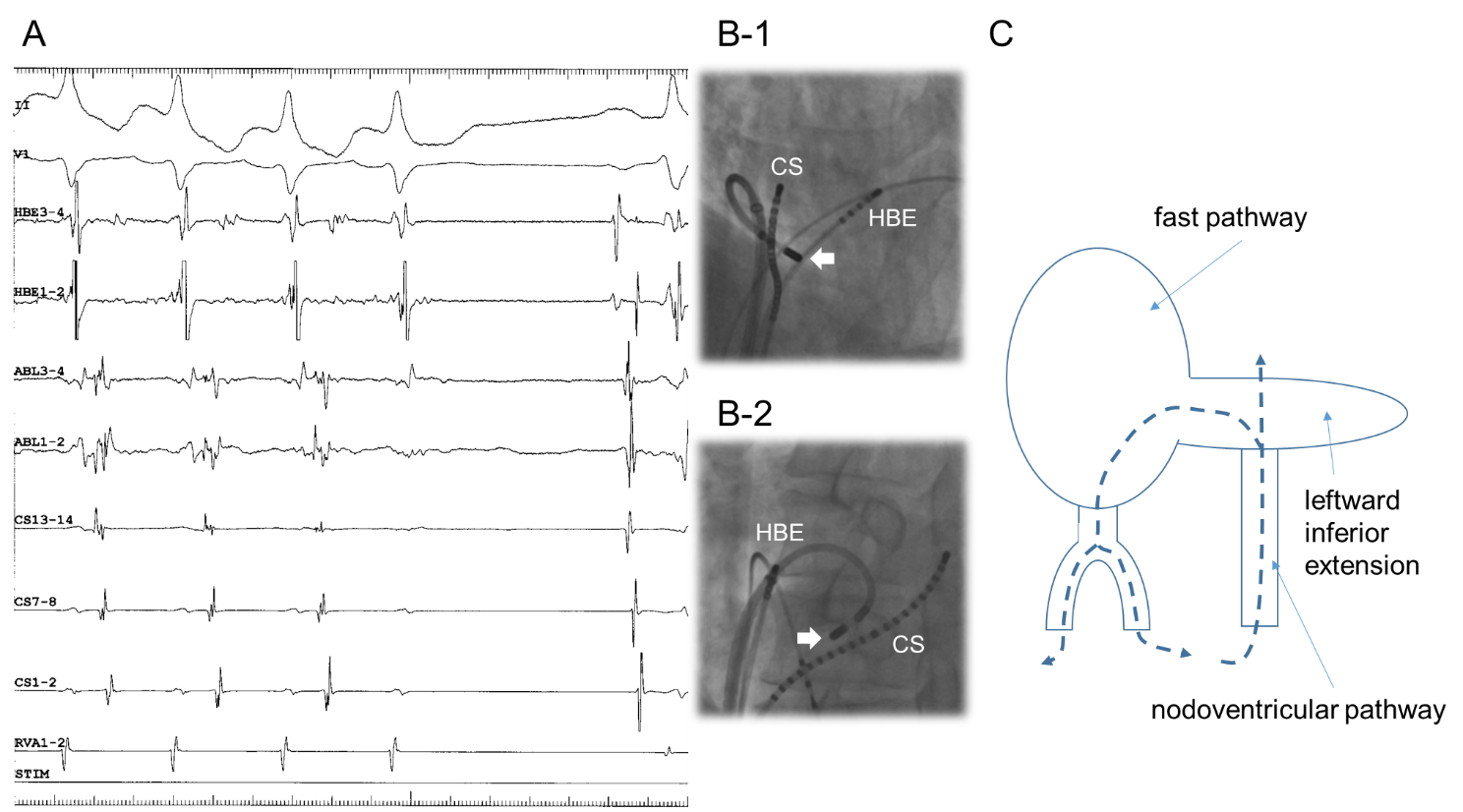
** **A:** Retrograde conduction over the nodoventricular pathway was eliminated during ongoing tachycardia by ablation at the site of earliest atrial activation. The tachycardia ended with ventricular activation (V no A termination) before resumption of sinus rhythm. ABL1-2 = distal and 3-4 = proximal recordings on the ablation catheter. See figure 1 for other abbreviations.

**B:** The site of successful ablation (white arrows) was in the left posterior septal region, along the mitral annulus, shown on the left (**B-1**) and right (**B-2**) anterior fluoroscopic projections.

**C:** This illustrates orthodromic reentrant tachycardia using a nodoventricular pathway with a connection to a left inferior AV nodal extension, a ventricular end away from mitral annulus and an atrial breakthrough in the left posteroseptum.

**Supplemental figure 4**

**
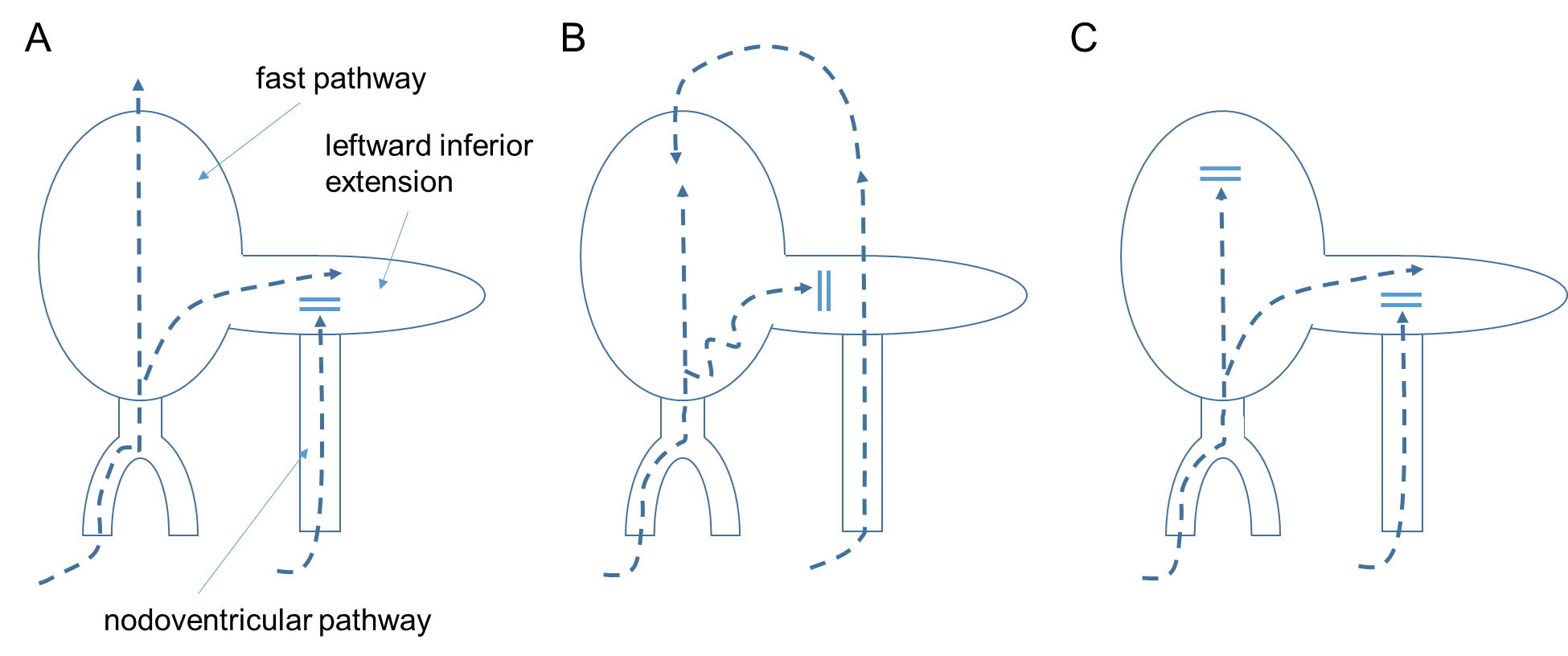
Putative illustration of retrograde atrial activation alternating between the fast and the nodoventricular pathways and during atypical 3:2 ventriculoatrial conduction**

**A**: After premature ventricular stimuli delivered a) during relatively slow ventricular stimulation (supplemental figure 1), or b) at a relatively long premature interval (figure 3A), the wavefront reaches the atria over the fast pathway, while simultaneously interfering with the atrial activation originating from the nodoventricular pathway.

**B:** If the retrograde premature wavefront interfering with the retrograde wavefront traveling over the nodoventricular pathway is delayed or blocked due to rapid stimulation (supplemental figure 2B) or short premature intervals (figure 3B), the retrograde wavefront traveling over the nodoventricular pathway can reach the atria without being interfered with and, eventually, may disturb the atrial breakthrough originating from the fast pathway.

Atypical 3:2 ventriculoatrial block (supplemental figure 2A) may result from a cycle-to-cycle, alternans of the interference described in **A** and **B.** In the 1^st^ cycle, preceded by ventriculoatrial block, retrograde atrial activation occurs over the fast pathway and blocks the atrial activation traveling over the nodoventricular pathway (**A**). In the 2^nd^ cycle, the retrograde wavefront traveling over the nodoventricular pathway is able to reach the atria due to frequency-dependent delay or block of the retrograde wavefront traveling over the fast pathway (**B**). In the 3^rd^ cycle, a) retrograde conduction over the fast pathway is blocked by the refractoriness left in the wake of its preceding penetration, and b) the attempt at atrial activation originating from the nodoventricular pathway is also blocked (**C**), resulting in complete ventriculoatrial block.
